# Procalcitonin and lung ultrasound algorithm to diagnose severe pneumonia in critical paediatric patients (PROLUSP study). A randomised clinical trial

**DOI:** 10.1186/s12931-020-01476-z

**Published:** 2020-10-08

**Authors:** Javier Rodríguez-Fanjul, Carmina Guitart, Sara Bobillo-Perez, Mònica Balaguer, Iolanda Jordan

**Affiliations:** 1 Neonatal Intensive Care Unit, Department of Paediatrics, Hospital Germans Trias i Pujol, Autonomous University of Barcelona, Badalona, Spain; 2 Paediatric Intensive Care Unit, Hospital Sant Joan de Déu, University of Barcelona, P° Sant Joan de Déu, 2, 08950 Esplugues, Barcelona, Spain; 3 Immunological and Respiratory Disorders in the Paediatric Critical Patient Research Group, Institut de Recerca Sant Joan de Déu, University of Barcelona, Barcelona, Spain; 4 Paediatric Infectious Diseases Research Group. Institut de Recerca Sant Joan de Déu, CIBERESP, Barcelona, Spain

## Abstract

**Background:**

Lung ultrasound (LUS) in combination with a biomarker has not yet been studied. We propose a clinical trial where the primary aims are: 1. To assess whether an algorithm with LUS and procalcitonin (PCT) may be useful for diagnosing bacterial pneumonia; 2. To analyse the sensitivity and specificity of LUS vs chest X-ray (CXR).

**Methods/design:**

A 3-year clinical trial. Inclusion criteria: children younger than 18 years old with suspected pneumonia in a Paediatric Intensive Care Unit. Patients will be randomised into two groups: Experimental Group: LUS will be performed as first lung image. Control Group: CXR will be performed as first pulmonary image. Patients will be classified according to the image and the PCT: a) PCT < 1 ng/mL and LUS/CXR are not suggestive of bacterial pneumonia (BN), no antibiotic will be prescribed; b) LUS/CXR are suggestive of BN, regardless of the PCT, antibiotic therapy is recommended; c) LUS/CXR is not suggestive of BN and PCT > 1 ng/mL, antibiotic therapy is recommended.

**Conclusion:**

This algorithm will help us to diagnose bacterial pneumonia and to prescribe the correct antibiotic treatment. A reduction of antibiotics per patient, of the treatment length, and of the exposure to ionizing radiation and in costs is expected.

**Trial registration:**

NCT04217980.

## Background

Pneumonia is responsible for more than 2 million child deaths around the world; it is the leading cause of childhood morbidity and mortality [1]. Moreover, severe lower respiratory infection is the most common cause of admission to paediatric intensive care units (PICUs) [2]. Around 60–70% of these have a viral etiology [3]. For physicians, it is important to differentiate between viral and bacterial infection because each entails a different therapeutic approach (whether antibiotics are indicated and the treatment duration), a different prognosis in the short and long term, and requires a different hospital isolation policy [4, 5]. It is challenging to rule whether a case of pneumonia is viral or bacterial in origin [6–8]. Clinical symptoms are similar and although conventional chest radiographs (CXR) have traditionally been considered the best diagnostic option [9], the specificity of CXR for determining bacterial etology remains low. Chest computed tomography may detect smaller pulmonary consolidations not visible via CXR, but it cannot be routinely performed on critically ill patients due to cost, exposure to radiation, and transportation risk [9]. Therefore, there is a need to have an examination tool that can be used at the patient’s bedside and which is easily reproducible to help detect lung consolidations. LUS has recently emerged as a radiation-free technique; it is non-invasive, with a high interobserver [10] agreement for lung pathologies such as consolidation [11], pleural effusion [12], interstitial syndrome [13], and pneumothorax [14]. To determine the etiology, conventional microbiology tests such as blood culture, pleural aspiration, and bronchoalveolar lavage are usual practices, but some of these are invasive, may not detect all etiologies [15], and the results may not be immediate.

Besides this, the use of biomarkers such as procalcitonin (PCT) has become more widespread during the past 10 years, helping clinicians diagnose bacterial etiology, especially in patients who have only had a fever for a few hours or those admitted to intensive care units [16–20]. The use of PCT has allowed for a decrease in antibiotic prescription [21, 22], even in nosocomial and community pneumonia [23, 24]. Despite its role in the diagnosis of pneumonia, PCT values without any other tests may not be a complete diagnostic biomarker for pneumonia.

Quality of care is defined by the World Health Organization as medical care in which the patient is diagnosed and treated correctly, according to current medical knowledge (scientific and technical quality), and to their biological factors (optimal state of health to attain), with a minimum cost (efficiency), minimum possible exposure to risk for more harm, and maximum patient satisfaction [25]. At the hospital level, a quality assurance plan should include different levels of action. The first step is level 1 of quality promotion, which requires institutional support and the availability of clinical and quality protocols. The second step is the research level: descriptive studies for detecting and quantifying a specific situation or health issue, and the use of databases or specific studies to evaluate health services, among others.

Therefore, we propose this clinical trial, based on combining LUS and PCT in an algorithm with the aim to improve quality of care in children with pneumonia in a PICU. We hypothesize that the diagnostic performance of LUS and PCT will be better than conventional CXR.

## Methods and design

The study is designed as a randomized, blinded clinical trial of children with severe community or nosocomial pneumonia. It will be conducted at a single PICU at Sant Joan de Déu Hospital, a tertiary children’s hospital in Barcelona. Period of recruitment and follow-up was from September 2017 to December 2019.

### Study aim

Our primary goal is to improve the quality of care in children with suspected community or nosocomial pneumonia in a PICU.

The main objectives are:

1. To assess whether a diagnostic algorithm for pneumonia that combines LUS and PCT may be useful in indicating and determining the duration of antibiotic treatment.
2. To analyse the sensitivity and specificity of LUS, compared to CXR, for severe community or nosocomial pneumonia.

The secondary aims are:

1. To quantify the irradiation dose avoided using LUS to replace CXR, and to determine if there is an associated decrease in costs.
2. To analyse interobserver agreement in LUS

### Inclusion and exclusion criteria

Inclusion: Children under 18 years old with suspected community pneumonia who require admission to the PICU or patients with suspected nosocomial pneumonia during their PICU stay.

Exclusion: Patients with underlying pathologies such as cystic fibrosis or who are immunocompromised. Patients who develop nosocomial pneumonia after being included in the study due to community pneumonia. Patients who have a CXR taken before being admitted to the PICU.

Withdrawal and abandonment criteria: Violation of study protocol, withdrawal of parental consent, death.

Patients withdrawing or with loss of protocol adherence will be excluded from the study.

### Definitions

- Community pneumonia: Patients with compatible clinical suspicion (fever, cough, tachypnoea, shortness of breath, abnormal respiratory auscultation sounds, hypoventilation, tubular breath sounds, thoracic or abdominal pain), compatible CXR (lobar consolidation, airspace opacity, pleural effusion, bullae, etc.) or LUS, changes in blood with a C-reactive protein higher than 50 mg/L and/or PCT higher than 1 ng/mL.
- Nosocomial pneumonia: Based on the Clinical Pulmonary Infection Score (CPIS) (Table 1).
- Ventilator-associated pneumonia: defined according to the Center for Disease Control criteria (CDC).

### LUS procedure

LUS will be performed by any of the 8 intensive care physicians who have received standard training in LUS (Winfocus PNCUS BL1P) and who have at least 3 years of experience using it. Team sessions focusing on the diagnosis of pneumonia with LUS will be repeated every 3 months to ensure quality and consistency in the LUS exam. The supplemental data included in the Shah et al. article will be used [26].

Subjects will be examined while they are in supine position. Imaging will be performed using a portable ultrasound device (Toshiba® Xario 200). A 12-MHz linear or a 5-MHz convex probe will be used, depending on the weight or size of the patient. A scan will be taken systematically in 3 areas for each hemithorax (anterior, lateral, and posterior), according to international recommendations [27]. Each area will be examined longitudinally and transversally.

In each area the following will be evaluated [27]: A-lines, B-lines (number and distance between them), lung sliding (M-mode), pleural space, lung consolidations, small subpleural consolidations, dynamic air bronchogram, vascular pattern, presence of lung point, and lung pulse.

The determination of a bacterial pneumonia ultrasound pattern will be based on the presence of lung consolidation with air bronchograms, which in initial stages are detected as small subpleural hypoechoic zones of less than 1 cm with bronchogram (not seen using conventional CXR) [28–31]. The determination of a viral pneumonia ultrasound pattern will be based on the presence of B-lines or coalescent B-lines with small subpleural consolidations of less than 1 cm, without bronchogram [32, 33].

### Methodology

The first thing to do will be to obtain consent from the parent(s) or legal guardian(s).

Patients who meet the inclusion criteria and sign the inform consent were randomly assigned to two groups using the “random” function in MS-Excel XP® program. A binary series of random numbers were generated according to the procedure described by Friedman. To procure a similar number of patients in both groups, the procedure created the sequence through a balanced block sampling. The series of numbers were held by the principal investigator and depending on the number of the patient tit was be assigned to on or another group. A total of 8 physicians enrolled participants, and the principal investigator assigned participants to interventions, depending on the randomized list. (Fig. 1)

- Experimental Group 1: The paediatrician-researcher (PR) performs the LUS at admission/suspicion as the first lung image test. If the paediatrician assistant (PA) requires a CXR, it can be performed, but the PR will not see the CXR. Patients will be subdivided into 3 groups:
  - If PCT is < 1 ng/mL and LUS is not suggestive of bacterial pneumonia (normal or viral), the patient will not receive an antibiotic.
  - If LUS is suggestive of bacterial pneumonia, regardless of PCT value, an antibiotic will be recommended.
  - If LUS is not suggestive of bacterial pneumonia, but PCT values are > 1 ng/mL, an antibiotic will be recommended to cover other infectious etiologies.
- Control Group 2: CXR will be performed as a first lung image test. Criteria to start an antibiotic will depend on the current unit protocol.
  - If PCT is < 1 ng/mL and CXR is not suggestive of bacterial pneumonia (normal or viral), the patient will not receive an antibiotic.
  - If CXR is suggestive of bacterial pneumonia, regardless of PCT value, an antibiotic will be recommended.
  - If CXR is not suggestive of bacterial pneumonia, but PCT value is > 1 ng/mL, an antibiotic will be recommended to cover other infectious etiologies.

Radiological and ultrasound patterns will be classified as: pneumonia (viral or bacterial), atelectasis, or parapneumonic pleural effusion (Table 2).

LUS will be performed every day following admission and recorded and stored. LUS images will be later analysed by a paediatric radiologist who is an expert in LUS and who has not seen the initial assessment and CXR, in order to evaluate interobserver agreement. CXR will be also be reported on by a paediatric radiologist consultant who has not seen the other results.

### Outcomes

The antibiotics protocol guided by LUS and PCT will be considered an improvement in the quality of care if a reduction in the prescription of antibiotics is observed, and also if there is a reduction in the number of days on antibiotics. Another primary outcome will be the increase in the sensitivity and specificity when diagnosing bacterial pneumonia using LUS. Secondary outcomes will be the reduction of the irradiation dose using the new protocol (with a reduction in economic costs as well), and a high LUS interobserver agreement.

### Data collection

The study coordinator will register patients, verifying compliance with all the inclusion criteria. An external company will be appointed to monitor the study and ensure compliance with correct clinical practice principles (ICHE6). Once the notebooks are audited, they will be entered into a validated database, one with restricted access by user level, which is equipped with inconsistency detection filters, and which affords data traceability until the database is no longer needed.

Data access: All the physicians and clinical researchers involved in the study, the Ethics Committee, and the relevant health authorities will have access to the data.

### Sample size

Sample size calculations will be performed using the statistical program Ene 2.0®. The main variable will be the existence of differences between CXR and LUS in patients with community pneumonia and nosocomial pneumonia. H1 will be considered as the existence of differences between LUS and CXR. An 80% power will be required to detect differences in the contrast of the null hypothesis H0: p1 = p2, using a bilateral X^2^ test for two independent samples. If we consider a significance level of 5%, it will be necessary to include 63 units in the control group and 63 units in the experimental group. A total of 126 patients will be included for community pneumonia. Twenty-eight patients per group will be included for nosocomial pneumonia; therefore, there will be 56 patients in total for this kind of pneumonia. After 12 months of recruitment, a preliminary analysis will be carried out to guarantee the safety of the patients. Respiratory infection represents around 30–70% of the PICU admissions, depending on the season. There will be an estimated 200–300 recruitable patients per year, so it is expected that the calculated sample size will be attainable.

### Statistical analysis

Using the “Random” function of the MS-Excel XP® program, a binary series of random numbers will be generated, according to the procedure described by Friedman [34]. This procedure creates the sequence by means of balanced block sampling to ensure a similar number of patients in each group. The series of numbers will be in the possession of the PICU’s head researcher. Depending on that number, each patient will be assigned to one group or the other. If a patient is randomized but does not complete the treatment, their data will not be analysed, and their random number will not be reused.

The categorical variables will be compared using the Chi-square test. The quantitative analysis will be compared using Student’s t-test or the Mann-Whitney U test, depending on whether the sample follows a normal distribution or not. A multivariate logistic regression analysis will be performed on those variables with statistical significance or a clear tendency in the univariate analysis to detect which factors represent a protective factor or not, in terms of quality. The analysis of the interobserver agreement will be performed using Cronbach’s alpha. A *p* < 0.05 will be considered significant. The statistical program SPPS® 20.0 will be used.

## Discussion

Nowadays, care standards are focused on the quality of care. As the World Health Organization stipulates, the patient must be diagnosed and treated correctly. This clinical trial is focused on improving the quality of care for paediatric patients with suspected bacterial pneumonia. LUS has good diagnostic accuracy for pneumonia in children, even if the exam is performed by a nonexpert physician [35]. Our algorithm will help us to diagnose bacterial pneumonia accurately, and to prescribe the correct antibiotic treatment. A reduction in patients on antibiotics and in the number of days on antibiotics is expected. Secondarily, a reduction in exposure to ionizing radiation and in costs is expected.

For many years, LUS has been integrated into the management of critically ill paediatric and neonatal patients at our hospital. Some articles have been published by our group regarding the use of LUS in different pathologies, such as prematurity, pulmonary arterial hypertension, during the postoperative period after cardiopulmonary bypass, etc. [36–38]. For the application of this clinical trial, all the researchers will be intensivists who are experts in LUS, and regular internal training will be essential to guarantee objective results.

In addition, our group has extensive experience in the use of PCT for the diagnosis of bacterial infection in other medical situations, such as after cardiopulmonary bypass in children and new-borns [19, 39]. Furthermore, we have experience in PCT-guided antibiotic policy, with a reduction in the number of days on antibiotics without adverse events in children with nosocomial infections [40] and in children after cardiopulmonary bypass [22].

## Conclusion

The use procalcitonin and lung ultrasound algorithm will help us diagnose bacterial pneumonia accurately and prescribe the correct antibiotic treatment. A reduction in patients on antibiotics and in a reduction in exposure to ionizing radiation and in costs is expected. This clinical trial is focused on improving the quality of care for paediatric patients with suspected bacterial pneumonia.

## Supplementary information

**Additional file 1 Supplemental Table 1.** Clinical Pulmonary Infection Score.

**Additional file 2 Supplemental Table 2.** Summary of the main findings using chest X-rays and lung ultrasound for diagnosing pneumonia.

**Additional file 3 Supplemental Figure 1.** Study protocol diagram.

## Data Availability

Not applicable.

## Abbreviations

LUS: Lung ultrasound
PCT: Procalcitonin
CXR: Chest X-ray
BN: Bacterial pneumonia
PICU: Pediatric intensive care unit
CPIS: Clinical Pulmonary Infection Score
CDC: Center for Disease Control Criteria
