# Supplementary material for: Procalcitonin and lung ultrasound algorithm to diagnose severe pneumonia in critical paediatric patients (PROLUSP study). A randomised clinical trial
Source: Respir Res. 2020 Oct 8;21:255. doi: 10.1186/s12931-020-01476-z (PMC7543673; doi:10.1186/s12931-020-01476-z)
Supplement: Supplementary file 1 — Additional file 1 Supplemental Table 1. Clinical Pulmonary Infection Score. [file 12931_2020_1476_MOESM1_ESM.pdf]

Supplemental table 1. Clinical Pulmonary Infection Score.

**CPIS points**	**0**	**1**	**2**
**Tracheal secretions**	Rare	Abundant	Purulent
**Chest X-ray**	No infiltrates	Diffuse infiltrate	Localized infiltrate
**Temperature (ºC)**	≥36.5 and ≤38.4	≥38.4 and ≤38.9	≤36 or ≥39
**Leukocytes (per mm^3)**	≥4,000 and ≤11,000	<4,000 or >11,000	<4,000 or >11,000 plus band forms ≥500
**PaO2/FiO2 ratio**	≥240 or ARDS	-	≤240 and no ARDS
**TA culture**	Negative	-	Positive

ARDS: Acute Respiratory Distress Syndrome. CPIS: Clinical Pulmonary Infection Score. TA: tracheal aspirate.
