# Supplementary material for: Procalcitonin and lung ultrasound algorithm to diagnose severe pneumonia in critical paediatric patients (PROLUSP study). A randomised clinical trial
Source: Respir Res. 2020 Oct 8;21:255. doi: 10.1186/s12931-020-01476-z (PMC7543673; doi:10.1186/s12931-020-01476-z)
Supplement: Supplementary file 2 — Additional file 2 Supplemental Table 2. Summary of the main findings using chest X-rays and lung ultrasound for diagnosing pneumonia. [file 12931_2020_1476_MOESM2_ESM.pdf]

Supplemental table 2. Summary of the main findings using chest X-rays and lung ultrasound for diagnosing pneumonia.

**Radiological findings in pneumonia** (Cherian T, Bull World Health Organ. 2005;83(5):353-359)
-End-point consolidation: Dense or spongy opacity involving a portion or whole of a lobe, or the entire lung; it may or may not contain air bronchograms.<br>-Infiltrate: Lineal with irregular densities (interstitial infiltrate) in a lacy pattern involving both lungs, with peribronchial thickening and multiple areas with atelectasis.<br>-Pleural effusion: Presence of fluid in the pleural space between the lung and the thoracic wall.
**Lung aeration ultrasound patterns** (Bouhamed B, Am J Resp Crit Care Med 2011;3:341-347)
1- Normal: A-lines with lung sliding and less than 2 isolated B-lines.<br>2- Moderate loss of aeration: Multiple, well-defined B-lines.<br>3- Severe loss of aeration: Multiple, coalescent B-lines.<br>4- Lung consolidation: Presence of tissue-like sign or shred sign.
**Lung pneumonia patterns** (Shah VP, JAMA Pediatr. 2013;167(2):119-125)
1. Bacterial pneumonia: Lung consolidation with bronchogram or small hypoechoic areas or subpleural consolidations with bronchogram.<br>2. Bronchograms are hyperechoic lineal areas inside lung consolidations.<br>3. Viral pneumonia: B-lines, confluent B-lines, small subpleural consolidations.<br>4. Pleural effusion: Anechoic space between parietal and visceral pleura or between the thoracic wall and diaphragm.<br>5. Atelectasis: Lung consolidation without bronchogram.<br>6. Pneumothorax: Presence of lung point.
