# Supplementary material for: Procalcitonin and lung ultrasound algorithm to diagnose severe pneumonia in critical paediatric patients (PROLUSP study). A randomised clinical trial
Source: Respir Res. 2020 Oct 8;21:255. doi: 10.1186/s12931-020-01476-z (PMC7543673; doi:10.1186/s12931-020-01476-z)
Supplement: Supplementary file 3 — Additional file 3 Supplemental Figure 1. Study protocol diagram. [file 12931_2020_1476_MOESM3_ESM.pdf]

Supplemental figure 1: Study protocol diagram.

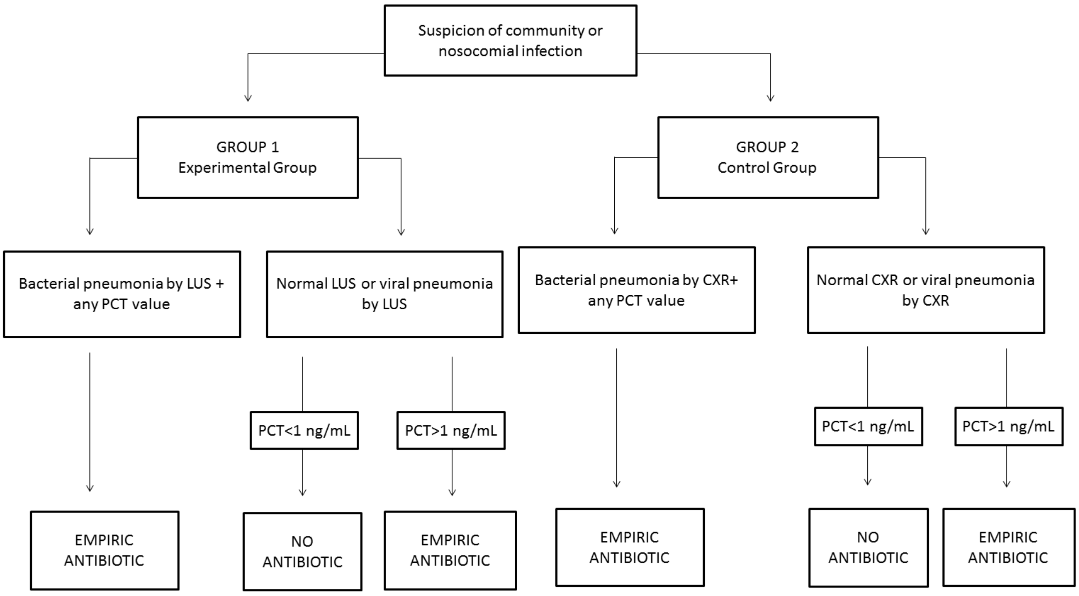

CXR: Chest X-ray; LUS: Lung Ultrasound; PCT: Procalcitonin.
